# The Susceptibility of *Bemisia tabaci* Mediterranean (MED) Species to Attack by a Parasitoid Wasp Changes between Two Whitefly Strains with Different Facultative Endosymbiotic Bacteria

**DOI:** 10.3390/insects14100808

**Published:** 2023-10-11

**Authors:** Massimo Giorgini, Giorgio Formisano, Rosalía García-García, Saúl Bernat-Ponce, Francisco Beitia

**Affiliations:** 1 Institute for Sustainable Plant Protection, National Research Council of Italy (IPSP-CNR), 80055 Portici, Italy; giorgio.formisano@ipsp.cnr.it; 2 Centro de Protección Vegetal y Biotecnología, Instituto Valenciano de Investigaciones Agrarias (IVIA), Moncada, 46113 Valencia, Spain; rosaliagarciagar@gmail.com (R.G.-G.); saulbernat2@gmail.com (S.B.-P.); beitia_fra@gva.es (F.B.)

**Keywords:** biological control, *Cardinium*, coevolution, host resistance, parasitism, parasitoid virulence, protective bacterial endosymbionts, *Eretmocerus mundus*, whiteflies

## Abstract

**Simple Summary:**

*Bemisia tabaci* is a complex of whitefly species, of which MEAM1 and MED species are highly invasive crop pests worldwide. The ecology of *B. tabaci* is affected by infections of facultative endosymbiotic bacteria, which may result in fitness benefits to their hosts, thus facilitating their adaptation to new environments. In some insects, endosymbionts protect their hosts from attack by parasitoid wasps by supplementing the host’s genetic defences. Parasitoids are effective biological control agents of *B. tabaci*. Understanding the variation in susceptibility to parasitization between whitefly strains harbouring different endosymbiotic bacteria is of practical importance. Here, we focused on MED species using two strains of the Q1 lineage, one infected with *Hamiltonella* and *Cardinium* (BtHC), and the other with *Hamiltonella* and *Rickettsia* (BtHR). We first saw no difference between BtHC and BtHR in parasitization by *Eretmocerus mundus* when reared on BtHC. On the other hand, BtHC nymphs were more resistant than BtHR after *E. mundus* were reared on BtHR for four generations. The same was observed after seven generations. Our findings showed that host strain is a factor affecting the ability of *E. mundus* to parasitize *B. tabaci* and support the hypothesis of *Cardinium* as a protective symbiont of the MED species. Moreover, our findings suggest that counteradaptations to *B. tabaci* defence mechanisms that maximize the parasitoid fitness may be rapidly selected in *E. mundus*.

**Abstract:**

In this study, two strains of the mitochondrial lineage Q1 of *Bemisia tabaci* MED species, characterized by a different complement of facultative bacterial endosymbionts, were tested for their susceptibility to be attacked by the parasitoid wasp *Eretmocerus mundus*, a widespread natural enemy of *B. tabaci*. Notably, the BtHC strain infected with *Hamiltonella* and *Cardinium* was more resistant to parasitization than the BtHR strain infected with *Hamiltonella* and *Rickettsia*. The resistant phenotype consisted of fewer nymphs successfully parasitized (containing the parasitoid mature larva or pupa) and in a lower percentage of adult wasps emerging from parasitized nymphs. Interestingly, the resistance traits were not evident when *E. mundus* parasitism was compared between BtHC and BtHR using parasitoids originating from a colony maintained on BtHC. However, when we moved the parasitoid colony on BtHR and tested *E. mundus* after it was reared on BtHR for four and seven generations, we saw then that BtHC was less susceptible to parasitization than BtHR. On the other hand, we did not detect any difference in the parasitization of the BtHR strain between the three generations of *E. mundus* tested. Our findings showed that host strain is a factor affecting the ability of *E. mundus* to parasitize *B. tabaci* and lay the basis for further studies aimed at disentangling the role of the facultative endosymbiont *Cardinium* and of the genetic background in the resistance of *B. tabaci* MED to parasitoid attack. Furthermore, they highlight that counteradaptations to the variation of *B. tabaci* defence mechanisms may be rapidly selected in *E. mundus* to maximize the parasitoid fitness.

## 1. Introduction

The whitefly *Bemisia tabaci* (Gennadius) (Hemiptera: Aleyrodidae) is one of the most invasive and injurious pests of a wide variety of crops worldwide, causing important economic losses due to direct plant damage and transmission of plant viruses [1,2,3]. *Bemisia tabaci* is a highly diverse complex of cryptic species, comprising about 40 entities differentiated by biological, behavioural, and ecological characters, and distinguishable based on molecular markers [4]. Most of these species are confined to specific geographic regions, while the Mediterranean (MED) species and the Middle East–Asia Minor 1 (MEAM1) species are highly invasive and are distributed worldwide, rising to the status of global pests [4,5,6,7]. The biology and ecology of *B. tabaci* strongly depend on symbiotic relationships with vertically inherited intracellular bacteria. The obligatory endosymbiont *Portiera aleyrodidarum* (the primary endosymbiont of all whitefly species) provides essential nutrients to the host and is necessary for its host development and reproduction [8,9]. In addition, single or multiple infections of facultative endosymbiotic bacteria of the genera *Hamiltonella*, *Arsenophonus*, *Wolbachia*, *Rickettsia*, *Cardinium*, *Hemipterophilus,* and *Fritschea* can be hosted by *B. tabaci* [10,11,12].

The interactions between facultative endosymbionts and arthropods are very diverse and affect host phenotypes dramatically [13]. Bacterial symbionts can act as reproductive manipulators, thus favouring the reproduction of infected females or as mutualists, resulting in a selective advantage for infected hosts [14,15]. Frequently, fitness benefits associated with facultative symbionts are context dependent, i.e., the symbionts are only beneficial if the stress factors against which they induce host protection (e.g., heat stress, natural enemies) are at play on the insect host population [16]. In the absence of the stress, the infection cost may exceed the positive effect on host fitness, leading to variation in infection frequency in host populations, in response to environmental variables even with seasonal fluctuations [17,18,19].

Facultative endosymbionts have important and contrasting ecological roles in *B. tabaci* [20,21]. Their infections may result in fitness benefits or costs, or even skew the host sex ratio toward females [22,23,24,25,26]. Indeed, facultative endosymbionts have been found to supplement *B. tabaci* with essential nutrients [27,28], confer tolerance to high temperatures [29,30], increase the resistance to entomopathogenic bacteria [31], manipulate the defence response of the host plant [32], and facilitate the transmission of geminiviruses to plants [33,34]. For invasive pests such as *B. tabaci* MED and MEAM1, the infection by facultative symbionts may accelerate the adaptation to new environments and intensify pest impact on agroecosystems. The community of bacterial endosymbionts is highly variable and is distinctive for different cryptic species of *B. tabaci*. Furthermore, it can also vary between different evolutionary lineages (mitochondrial variants) of a single species, as it is the case with the MED species [7,10,12,35,36,37]. Also, in natural populations of *B. tabaci*, infection frequency and phenotypic effect of endosymbionts can change over time and according to geographic location, host plants, and nuclear genotype [19,24,38,39].

Parasitoid wasps (Hymenoptera: Aphelinidae) are important natural factors limiting the populations of *B. tabaci*, frequently by acting in combinations with predatory species [40,41,42,43]. They are fundamental in implementing IPM strategies against *B. tabaci* and have been applied as classical biological control agents in invaded geographic areas or in augmentative and conservation biological control of *B. tabaci* worldwide [43,44,45]. Insect species can respond to the attack of parasitoids (and more, in general, of parasites and pathogens) with genetic defence mechanisms, such as the immune response, but they can also rely on the activity of protective microbial symbionts [46,47,48,49]. Likewise, parasitoids show considerable genetic variation in their virulence traits and, consequently, also display a high adaptive potential to genetic defence mechanisms [50,51] and protective symbionts [52] of their insect hosts.

*Eretmocerus mundus* Mercet is a parasitoid species native to the Mediterranean region (and distributed throughout the Palearctic region) where it is one of the most common and efficient parasitoids of *B. tabaci* MEAM1 and MED species [53,54,55]. Currently, *E. mundus* is distributed worldwide [56,57,58,59] and has become established into the USA, after its introduction for the classical biological control of *B. tabaci* [40]. Large differences in biology and behaviour have been found between geographical populations of *E. mundus* [60,61,62]. *Eretmocerus mundus* appears to have evolved a unique mechanism to escape the immune response of the host nymphs. First, the female wasp oviposits the egg ventrally and externally to the body of the host (second- or third-instar nymph) [63]. Second, as soon as the young larva starts the penetration into the body of the fourth-instar nymph, it induces the host to form a capsule of epidermal cells around itself [64,65]. As a result, the parasitoid larva becomes completely isolated from the host hemocoel and then from the host’s immune system. The capsule remains intact during most of the second larval instar of the parasitoid and then disintegrates; after that, the parasitoid larva feeds on the host’s tissues. Although *B. tabaci* appears highly susceptible to parasitization by *E. mundus*, the results of some studies suggest a role of protective symbionts in the defence mechanisms against the attack of parasitoids. In the MEAM1 species, the genes of *Rickettsia* symbionts responded strongly to parasitization by *E. mundus* and in parallel, symbiont cell proliferation was also observed in the whitefly body [66].

In this study, we focused on the Q1 mitochondrial lineage of the MED species of *B. tabaci*, which is endemic in the western Mediterranean and invasive worldwide [5,35]. Populations of MED Q1 are characterized by fixed or nearly fixed *Hamiltonella* infection. Coinfections of *Hamiltonella* with other facultative endosymbionts are common in most populations, with *Cardinium* and/or *Wolbachia* being the most frequent associates. The infection prevalence of *Cardinium* and *Wolbachia* is highly variable, ranging from very low to almost 100%, both in the native Mediterranean area and in invaded regions [35,36,37,67,68,69]. Instead, few or no individuals harbouring *Rickettsia* or *Arsenophonus* are generally present in Q1 populations. In MED Q1 individuals, *Hamiltonella* lost the genes that confer resistance to parasitoids in aphids [70], while they have conserved the genes involved in the synthesis of essential nutrients, thus having a nutritional role complementary to that of the primary symbiont [28,71]. The role of *Rickettsia* and *Arsenophonus* seems to be relatively irrelevant considering their overall low infection prevalence. While the function of *Wolbachia* is still unexplored, *Cardinium* appears to have a context-dependent effect on MED Q1. *Cardinium* symbionts confer fitness benefits to *B. tabaci* Q1 under conditions of thermal stress [30], while being costly for host fitness under normal temperature regimes [26,72,73], which can explain the fluctuating prevalence of infection within host populations and across different geographic regions. Furthermore, the sequencing of the *Cardinium* genome revealed the presence of genes encoding for proteins with potential insecticidal activity, suggesting the role of a defensive symbiont in MED Q1 [74]. More recently, *Cardinium* has been found to upregulate proteins related to the immune response of *B. tabaci* MED [71]. Overall, *Cardinium* appears to perform multiple functions in increasing the host’s adaptation to the environment and may represent an important factor in favouring the invasion process of *B. tabaci* MED Q1. Here, we tested the hypothesis that two strains of *B. tabaci* MED Q1 from different locations in southwestern Europe and with different infections of facultative endosymbionts could show different susceptibility to parasitization by *E. mundus,* and that the strain harbouring *Cardinium* was more resistant. The results obtained confirmed the hypothesis and highlighted that counteradaptations of *E. mundus* to the defence mechanisms of *B. tabaci* may be rapidly selected to maximize the fitness of the parasitoid.

## 2. Materials and Methods

### 2.1. Studied Insects

Two strains of the Q1 mitochondrial lineage of the MED species of *B. tabaci*, each characterized by a different complement of facultative endosymbionts, were established in the laboratory on cotton (*Gossypium hirsutum* L.) plants. The strain BtHC, characterized by a fixed infection of *Hamiltonella* and *Cardinium*, originated from a population collected on black nightshade (*Solanum nigrum* L.) plants from Moncada, Valencia, Spain in 2014. The strain BtHR, characterized by a fixed infection of *Hamiltonella* and *Rickettsia*, originated from a population collected on rose mallow (*Hibiscus moscheutos* L.) plants from Angers, France in 2012. The mitochondrial lineage of each whitefly strain was identified by restriction analysis of COI amplicons as described by the authors of [6,75]. To assess the composition of the facultative endosymbiont community, we used specific PCR primers targeting the 16S rRNA gene for *Cardinium*, *Hamiltonella*, *Rickettsia*, and *Wolbachia*, and the 23S rRNA gene for *Arsenophonus* [10].

**Figure 1:**
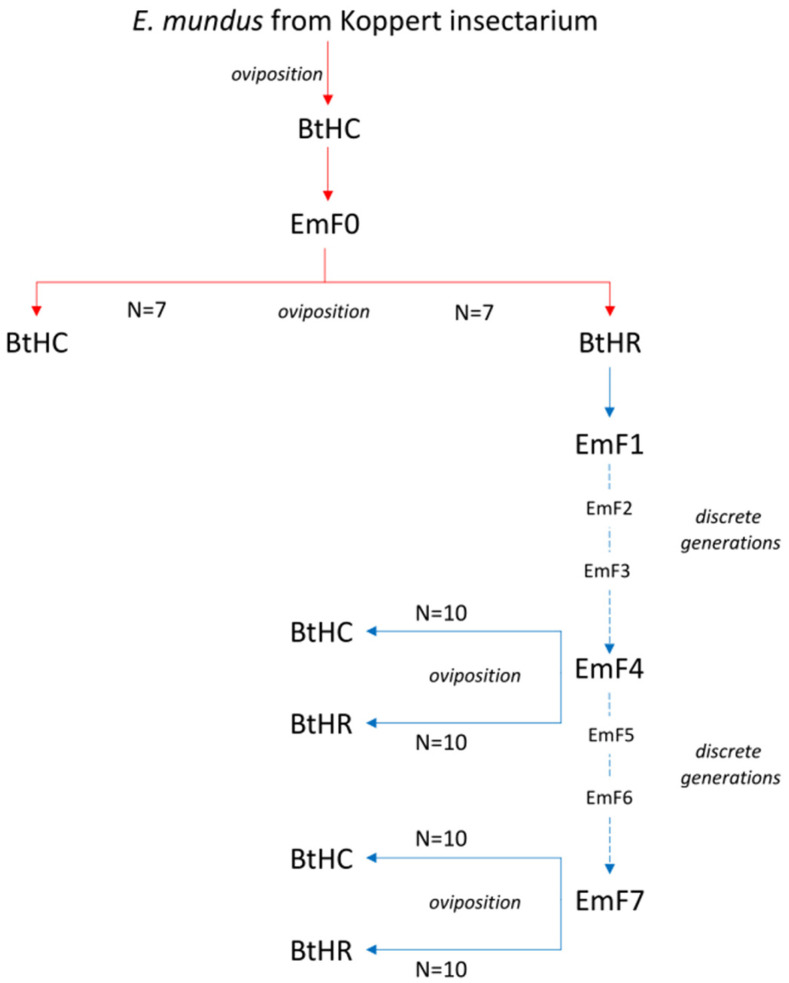
Experimental design. BtHC and BtHR are two strains of *Bemisia tabaci* of the lineage Q1 of the MED species infected by *Hamiltonella* and *Cardinium* and by *Hamiltonella* and *Rickettsia*, respectively. For each of the BtHC and BtHR strains, the parasitism of 7 EmF0, 10 EmF4, and 10 EmF7 *Eretmocerus mundus* females was assessed. EmF0 is the parental generation reared on BtHC; EmF4 and EmF7 are the fourth and the seventh generation of the parasitoid reared on BtHR, respectively, and used for bioassays.

*Eretmocerus mundus* individuals were first obtained in 2014 from Koppert Biological Systems, S.L. (Águilas, Murcia, Spain). The parasitoids were mass-reared in Koppert’s insectarium on a strain of *B. tabaci* MED Q1 infected by *Hamiltonella* and *Cardinium*. Before starting the bioassays, we reared the Koppert strain of *E. mundus* on BtHC for one generation. Subsequently, a colony of the parasitoid was established on BtHR, starting from individuals obtained from BtHC (Figure 1).

The two whitefly strains and the *E. mundus* colony were maintained at IVIA, Spain, in separate environmental chambers at 25 ± 1 °C, 60–70% relative humidity, and 16:8 photoperiod.

### 2.2. Response of Eretmocerus mundus Oviposition Rate to Host Density

Before evaluating the effect of the strain on the susceptibility of *B. tabaci* MED Q1 to *E. mundus* parasitism, we set up an experiment aimed at assessing the density of host nymphs that maximized the oviposition rate of *E. mundus* in our experimental arena. This latter consisted of a 6 cm diameter cotton leaf disk infested with whitefly nymphs and laid onto a 4–5 mm thick layer of 1% water agar in a 6 cm ventilated Petri dish. Densities of 25, 50, 75, and 100 third-instar nymphs were compared. To have the same age nymphs infesting each leaf disk, 100 adult whiteflies (~1:1 sex ratio) of the BtHC strain were allowed to oviposit for 24 h, confined in a 3.5 cm diameter clip cage, on the abaxial surface of a cotton leaf of a potted plant. After having removed the adult whiteflies, the leaf was detached from the plant when the nymphs were at the second/third instar and then the leaf disc was excised. After laying the leaf disc on the agar layer, excess nymphs were gently removed by a pin under a stereoscope to have a uniform spatial distribution of third-instar nymphs and no wounds to the leaf disc.

*Bemisia tabaci* pupal cases parasitized by *E. mundus* (from Koppert) were stored in a rearing cage and the freshly emerged adult parasitoids were collected by an aspirator after 24 h from the emergence so that females had a high chance to be mated in the cage. Then, single 24-h-old mated parasitoid females were introduced into an experimental arena. To allow for female re-mating and avoid the negative effect of sperm depletion on the oviposition rate, a 24-h-old male wasp was introduced into the arena together with the female wasp. It was possible to use 24-h-old females as no pre-oviposition period is required for *E. mundus* [55]. The parasitoid female was allowed to oviposit for 24 h, after which the whitefly nymphs were observed under a stereoscope (40× magnification) to count the eggs laid. Each whitefly nymph was lifted up from the leaf surface to check for the occurrence of the *E. mundus* egg underneath the body. Only when both female and male wasps were recovered alive at the end of the oviposition time was the experimental arena considered for fecundity measurement. Experimental conditions were 25 ± 1 °C, 60–70% relative humidity, and 16:8 photoperiod. Overall, the replicates were 9, 11, 9, and 9 females for each treatment of 25, 50, 75, and 100 nymphs/arena, respectively. We used one-way ANOVA to test if the mean numbers of eggs differed statistically among the four treatments. A post hoc test for multiple comparisons was run using Tukey’s HSD test at the 95% confidence interval.

### 2.3. Effect of Bemisia tabaci Strain on Parasitism by Eretmocerus mundus

In this experiment, given the presence in the *Cardinium* genome of genes coding for proteins with potential insecticidal activity [74], we hypothesized that the BtHC strain was more resistant to parasitization than the BtHR strain. To test this hypothesis, we set up a culture of *E. mundus* on the BtHR strain and evaluated the parasitoid activity comparatively on BtHC and BtHR. The maintenance of *E. mundus* on the BtHR strain was intended to reduce selection in favour of individuals with a greater virulence against the BtHC strain.

One hundred 24-h-old *E. mundus* mated females were selected from a batch of individuals supplied by Koppert and were inoculated on cotton plants infested by BtHC nymphs. The *E. mundus* progeny that emerged from BtHC nymphs was the parental generation (hereafter, named EmF0) in our bioassay. Single EmF0 females were allowed to oviposit for 24 h in an experimental arena infested by 100 third-instar nymphs of BtHC or BtHR, following the protocol described above. Then, the first-generation progeny of *E. mundus* reared on BtHR was used to establish a parasitoid colony on the same BtHR host strain by allowing the development of successive discrete generations. Single *E. mundus* females selected from the fourth generation (EmF4) were allowed to oviposit in an experimental arena with BtHC or BtHR nymphs. The same was performed using *E. mundus* females selected from the seventh generation reared on BtHR (EmF7). We predicted to find greater resistance by the BtHC strain than the BtHR strain when subjected to parasitization by EmF4 and EmF7 females, compared to EmF0 females reared on BtHC. Overall, for each of the BtHC and BtHR strains of *B. tabaci*, the parasitism of 7 EmF0, 10 EmF4, and 10 EmF7 females was assessed (Figure 1).

*Eretmocerus mundus* parasitism was determined by measuring two parameters, namely the number of parasitized whitefly nymphs (i.e., containing the parasitoid’s mature larva or pupa) and the parasitoid’s development success rate; the latter was estimated as the percentage of adult wasps emerged from parasitized nymphs. The parasitoid development within the host nymphs was checked eight–ten days after the parasitoid oviposition in the experimental arena. Nymphs containing fully developed parasitic larva or pupa were gently collected from the leaf disc and isolated individually in a vial, waiting for adult offspring.

As a measure of the development success of the *E. mundus* eggs, we compared the number of eggs laid by *E. mundus* females in 100 BtHC nymphs (see the experiment described above in Section 2.2) with the number of parasitized nymphs produced by EmF0 females ovipositing in BtHC or BtHR nymphs. Student’s *t* test at the 95% confidence level was used for the two pairwise comparisons.

The first instar of *E. mundus* induces permanent developmental arrest of the fourth-instar nymph before the penetration into the host body, resulting in the development arrest, and ultimately, the death of the attacked nymph [65]. Consequently, if the nymphs of one of the two strains of *B. tabaci* are less susceptible to the penetration of the parasitoid first-instar larva, they are expected to suffer higher mortality after the attack of the parasitoid larva. We compared the number of dead nymphs (without evidence of a parasitoid larva within) of BtHC and BtHR strains as an additional measure of the host’s susceptibility to the parasitoid. The count of dead nymphs on each arena continued until all healthy nymphs completed development and adult whiteflies emerged.

Analysis of data was performed by building generalized linear models using the parasitism parameters of *E. mundus* (number of nymphs containing the parasitoid’s mature larva or pupa, percentage of adult wasps emerged from parasitized nymphs, and number of dead nymphs) as dependent variables and including *B. tabaci* strain (BtHC vs. BtHR), parasitoid generation (EmF0 vs. EmF4 vs. EmF7), and their interaction as explanatory variables. The best model for each dependent variable was chosen based on the lowest value of Akaike information criterion statistics (AIC, AICC, and CAIC). The number of parasitized nymphs containing the *E. mundus* larva or pupa was fitted to a gamma distribution model with the identity link function. The number of dead nymphs was fitted to a Poisson distribution model using the identity link function. The proportion of adult wasps that emerged from parasitized nymphs was fitted to a binomial distribution model with the logit link function. The overall significance of each predictor and their interaction was assessed by Wald chi-squared statistics. Post hoc tests for multiple comparisons were run using Fisher’s LSD test with the Bonferroni method of adjustment (*p*-value adjusted to 15 pairwise comparisons between the six groups of data) at the 95% confidence interval. All statistical analyses were performed using IBM SPSS Statistics for Windows, Version 28.0.1.1 (IBM Corp., Armonk, NY, USA).

## 3. Results

### 3.1. Response of Eretmocerus mundus Oviposition Rate to Host Density

**Figure 2:**
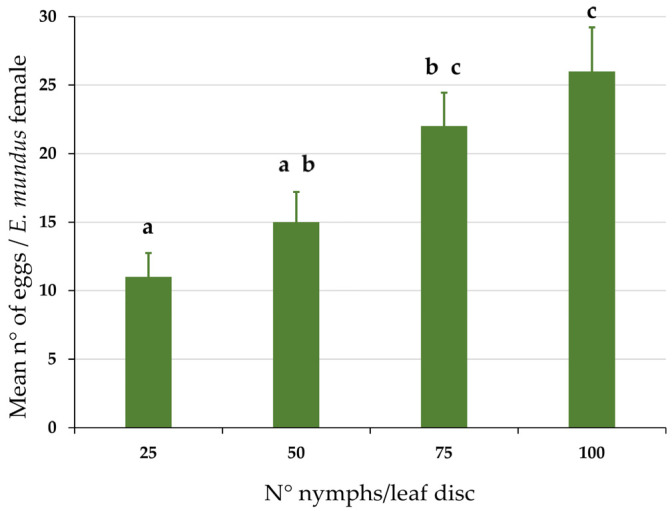
Mean number of eggs laid by *Eretmocerus mundus* females at different densities of *Bemisia tabaci* nymphs of the BtHC strain. Bars indicate standard error. Means bearing different letters are significantly different after Tukey’s HSD post hoc test at the 95% confidence level (25 vs. 75 nymphs *p* = 0.024; 25 vs. 100 nymphs *p* = 0.001; 50 vs. 100 nymphs *p* = 0.017).

The density of BtHC nymphs significantly influenced the fecundity of *E. mundus*. The number of eggs oviposited by single females increased as the number of the host nymphs increased (one-way ANOVA, F_3,34_ = 7.12, *p* = 0.001) (Figure 2). However, the mean number of eggs/parasitoid female did not differ significantly between host densities of 75 and 100 nymphs/arena. Considering the greater ease in selecting the right number of host nymphs, we decided to use the density of 100 nymphs/arena to set up the experiment, aiming at evaluating the effect of the *B. tabaci* strain on *E. mundus* parasitism.

### 3.2. Effect of Bemisia tabaci Strain on Parasitism by Eretmocerus mundus

**Figure 3:**
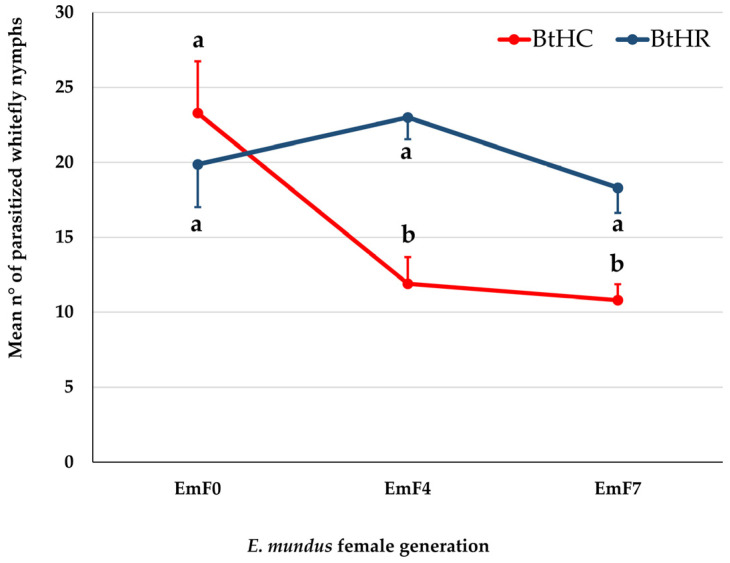
Mean number of *Bemisia tabaci* nymphs of the strain BtHC or BtHR parasitized by *Eretmocerus mundus* females of the parental (EmF0), fourth (EmF4), or seventh (EmF7) generation. Different letters indicate significantly different means after a GLM analysis followed by Fisher’s LSD post hoc test with Bonferroni correction (adjusted *p*-values in the text). Bars indicate standard error.

The GLM model showed a significant effect of host strain (Wald χ^2^ = 8.82, d.f. = 1, *p* < 0.001), parasitoid generation (Wald χ^2^ = 10.87, d.f. = 2, *p* = 0.004), and their interaction (Wald χ^2^ = 10.07, d.f. = 2, *p* = 0.006) on the number of nymphs parasitized by *E. mundus*. A multiple comparison analysis showed that BtHC nymphs were significantly less parasitized than BtHR nymphs when exposed to EmF4 (*p* = 0.0005) and EmF7 (*p* = 0.009) females, while no difference between the two strains emerged when parasitization was made by EmF0 females (Figure 3). Furthermore, the number of parasitized nymphs of the BtHR strain did not differ significantly among the three generations of *E. mundus*. Conversely, when *E. mundus* was allowed to oviposit on the BtHC strain, the number of nymphs parasitized by EmF0 was significantly higher than the number of nymphs parasitized by EmF4 (*p* = 0.007) or EmF7 (*p* = 0.001).

The number of eggs laid by *E. mundus* females in 100 BtHC nymphs (mean = 26 ± 2.51 s.e.) (Figure 2) was not significantly different from the number of nymphs parasitized by EmF0 females (Figure 3), both when the host was BtHC (mean = 23.29 ± 2.87 s.e.; t = 0.57, d.f. = 14 and *p* = 0.58) and BtHR (mean 19.86 ± 2.44 s.e.; t = 1.38, d.f. = 14 and *p* = 0.19) (Student’s *t*-test at the 95% confidence level). This result suggests that most of the eggs laid by EmF0 females gave rise to the complete development of a parasitoid larva.

**Figure 4:**
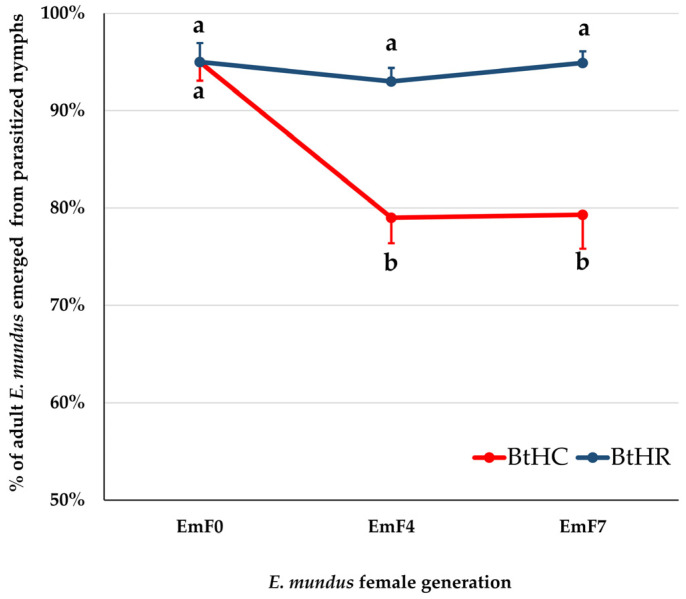
Proportion of adult wasps that emerged from *Bemisia tabaci* nymphs of the strains BtHC or BtHR parasitized by *Eretmocerus mundus* females of the parental (EmF0), fourth (EmF4), or seventh (EmF7) generation. Different letters indicate significantly different means after a GLM analysis followed by Fisher’s LSD post hoc test with Bonferroni correction (adjusted *p*-values in the text). Bars indicate standard error.

The GLM binomial model showed a significant effect of host strain (Wald χ^2^ = 11.08, d.f. = 1, *p* = 0.001), parasitoid generation (Wald χ^2^ = 6.87, d.f. = 2, *p* = 0.03), and their interaction (Wald χ^2^ = 8.65, d.f. = 2, *p* = 0.01) on the proportion of adult wasps that emerged from parasitized nymphs. Multiple comparison analysis showed (Figure 4) that the proportion of adult wasps emerged from nymphs of the BtHR strain was significantly higher than that observed on the BtHC strain, both when the nymphs were parasitized by EmF4 females (*p* = 0.01) and by EmF7 females (*p* = 0.01). However, there was no significant difference between host strains when nymphs were parasitized by EmF0 females. Furthermore, the proportion of adult wasps that emerged from parasitized BtHR nymphs did not vary significantly between the three generations of *E. mundus*. Conversely, when parasitoid development occurred in BtHC nymphs, the proportion of adult wasps emerged from nymphs parasitized by EmF0 females was significantly higher than that obtained from nymphs parasitized by EmF4 (*p* = 0.002) or EmF7 (*p* = 0.014) females (Figure 4).

**Figure 5:**
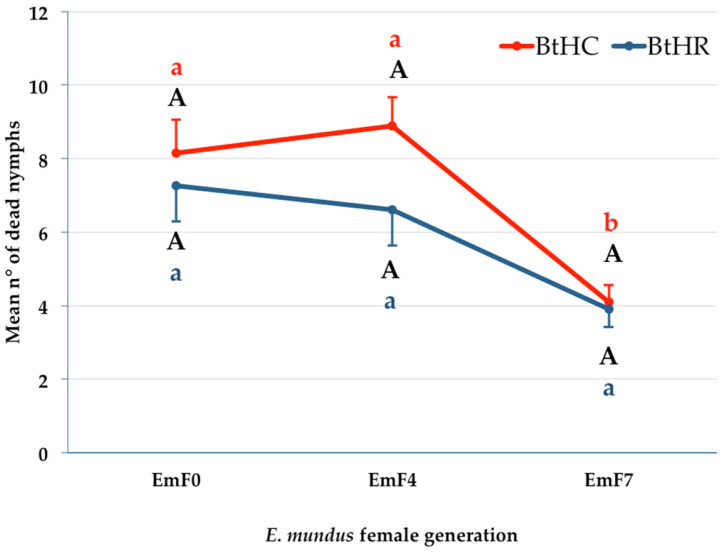
Mean number of dead nymphs of *Bemisia tabaci* of strains BtHC or BtHR observed in experimental arenas treated with *Eretmocerus mundus* females of the parental (EmF0), fourth (EmF4), or seventh (EmF7) generation. For the BtHC strain, different red lowercase letters indicate significantly different means after a GLM analysis followed by Fisher’s LSD post hoc test with Bonferroni correction (adjusted *p*-values in the text). For the BtHR strain, means do not differ significantly between the three generations of *E. mundus* (identical blue lowercase letters). Means do not differ significantly in pairwise comparisons between the two strains (identical black capital letters). Bars indicate standard error.

The GLM model revealed that there was no statistically significant interaction effect of host strain and parasitoid generation on the number of dead nymphs (Wald χ^2^ = 1.88, d.f. = 2, *p* = 0.39). However, while the host strain had no effect on the number of dead nymphs (Wald χ^2^ = 2.47, d.f. = 1, *p* = 0.12), there were significant differences between the *E. mundus* generations (Wald χ^2^ = 32.64, d.f. = 2, *p* < 0.001). Multiple comparison analysis showed that for the BtHR strain, there were no significant differences in the number of dead nymphs between the three generations of *E. mundus*. For the BtHC strain, the number of dead nymphs observed in arenas treated with EmF7 females was significantly lower than that observed for EmF0 (*p* = 0.02) and EmF4 (*p* = 0.0004) females (Figure 5).

## 4. Discussion

In this study, we tested the hypothesis that the host strain may be a factor affecting the ability of *E. mundus* to parasitize the whitefly *B. tabaci*. Focusing on two strains of the Q1 mitochondrial lineage of *B. tabaci* MED species, each one characterized by a specific set of facultative endosymbionts, we showed that the whitefly strain BtHC harbouring the bacteria *Hamiltonella* and *Cardinium* was more resistant to parasitoid attack than the strain BtHR infected by *Hamiltonella* and *Rickettsia.* The resistant phenotype of the BtHC strain, compared with the BtHR strain, consisted of fewer parasitized nymphs hosting the fully developed larva or pupa of the parasitoid and a lower parasitoid’s development success rate (percentage of adult wasps emerged from parasitized nymphs). To explain the difference between BtHC and BtHR host strains in the overall number of nymphs parasitized by *E. mundus*, we suggest two hypotheses. The first one is that female wasps laid fewer eggs on BtHC nymphs. The second one is that *E. mundus* suffered greater mortality during the stages of egg or early larval instar when the latter penetrates the nymph. Although we did not set up a parallel bioassay to assess the number of eggs laid by *E. mundus* on the two whitefly host strains, some reasons make the second hypothesis less reliable. First, we found that most of the eggs laid by *E. mundus* parental females (EmF0) gave rise to the complete development of a parasitoid larva regardless of whether the oviposition occurred in the BtHC or BtHR nymphs. Furthermore, in consideration of the oviposition behaviour of *E. mundus*, it is unlikely that the eggs suffered a different mortality rate between BtHC and BtHR. *Eretmocerus mundus* females lay the eggs (most frequently one egg/nymph) externally and ventrally to the body of second- and third-instar nymphs of *B. tabaci* [63]. Thus, the host’s immune system or endogenous toxins cannot target the egg. After the first-instar larva of *E. mundus* emerges, it penetrates the host nymph only when the latter reaches the fourth instar [65]. The start of larva penetration induces the host to form a capsule composed of epidermal cells around the parasitoid larva, which is consequently completely isolated from the host tissues. The capsule function is not well understood and could be that of a nutritional mediator between host and parasitoid and/or that of hindering direct contact between the larva and the host’s immune system. The capsule remains intact during most of the second larval instar of the parasitoid and then disintegrates, after which, the parasitoid larva feeds on the host’s tissues [63,64]. In addition, starting from the pre-penetrating stage, the parasitoid larva is able of inducing permanent developmental arrest in the fourth-instar nymph, which will no longer produce an adult whitefly [65]. Consequently, if the first-instar larva ingests toxins and dies during penetration, we should expect the host nymph to die as well. Extending to our study, if the nymphs of one of the two strains of *B. tabaci* are less susceptible to the full penetration of parasitoid first-instar larva, these nymphs are expected to experience increased mortality. This was not the case. We found that the host strain has no effect on the number of dead nymphs (without evidence of a parasitoid larva within), indicating that there are no differential mortality factors between BtHC and BtHR during the early stages of parasitoid larval development. Overall, our data suggest that nymphs of the BtHC strain may likely be less preferred by *E. mundus* for oviposition than nymphs of the BtHR strain. In addition, we showed higher parasitoid mortality during the mature larva/pupa instar in BtHC nymphs. This is in accordance with the feeding behaviour of the parasitoid larva, which begins to feed on the host’s tissues from the second larval instar when it could come into contact with or ingest toxins present in the host.

The observed difference in *E. mundus* parasitism between the two strains of *B. tabaci* could depend on two factors, namely the host’s genetic background and the defensive phenotype of the host’s bacterial symbionts [76]. Most experiments performed to quantify the effect of the two components on the expression of the defensive phenotype have shown that intra-specific variation for resistance to parasites (i.e., parasitoids, entomopathogenic fungi or nematodes) is mostly due to variation in the infection by defensive endosymbionts rather than genetic variation among strains [49]. Although we compared two closely related strains of *B. tabaci* MED species (both strains were of the Q1 mitochondrial genetic variant), we cannot exclude an impact of the host genotype on the performance of *E. mundus*, as our experimental design was not able to separate the effect of host genotype and symbiont-induced protection. However, our finding of a resistant phenotype associated to the BtHC strain, infected by *Hamiltonella* and *Cardinium*, compared to the BtHR strain, infected by *Hamiltonella* and *Rickettsia*, supports the results of a previous study about a possible role of *Cardinium* as a defensive symbiont of the Q1 lineage of *B. tabaci* MED species [74]. The authors, by sequencing and analysing the entire genome of the symbiont, ruled out important functions of *Cardinium* in host nutrition, but found genes encoding proteins with potential insecticidal activity. Because these genes were highly expressed, the authors of [74] suggested an important and beneficial function of these genes for the host. The insecticidal activity of *Cardinium* toxins may be favoured by the tissue tropism of the symbionts. The latter, in addition to living in bacteriocytes, are distributed throughout the body of the whitefly [74,77]. Consequently, *Cardinium* bacteria can come easily in contact with a parasitoid egg or larva and may kill them by secreting toxins that can affect a parasitoid directly or after the bacteria have invaded the parasitoid body [74]. Considering the parasitization behaviour of *E. mundus*, *Cardinium* toxins could target only the parasitoid larva, probably only during the final part of its development, after it has been freed from the protective capsule. Indeed, we found a lower parasitoid development success rate in the BtHC strain than in the BtHR strain. Additionally, *Cardinium* infection upregulates proteins related to the immune response of *B. tabaci* MED [71]. However, it is less likely that such a mechanism could act against *E. mundus*, as the protective capsule shades the parasitoid young larva from the host immune response.

Of the two defensive responses associated with *Cardinium* infection in the BtHC strain of *B. tabaci*, the death of the parasitoid larva in the host body is the physiological response observed in all studied cases of symbiont-induced defence against parasitoids, which include examples in aphids [46,78,79] and *Drosophila* flies [80,81,82]. Conversely, a change in the oviposition behaviour of female wasps between infected and uninfected hosts was not generally observed [46,80,83]. However, in some cases, aphids infected by *Hamiltonella defensa* [84] or *Serratia symbiotica* [85] resulted less attractive to parasitoid females, which preferred to oviposit in uninfected hosts. Facultative endosymbionts, including the defensive ones, alter the host’s metabolic capacity as an effect of competition with hosts for nutrients [86]. Parasitoids are able to perceive variations in the host’s metabolite profile induced by defensive endosymbionts, including variations in sex pheromones [87] or other volatile compounds [85], cuticular hydrocarbon profiles, and honeydew composition [86]. Parasitoids of whiteflies rely on chemical signals from the host’s honeydew and cuticula to recognize their host nymphs [88,89]. *Cardinium* could alter the host’s chemical profile that regulates the foraging behaviour of parasitoids to make *B. tabaci* MED nymphs less susceptible to parasitization.

A further result of this study was that the effect of the host strain on *E. mundus* increased with the succession of generations of the parasitoid. Indeed, the resistant phenotype associated with BtHC became evident after *E. mundus* was tested on BtHC after four and seven generations of rearing on BtHR, while no difference emerged between BtHC and BtHR parasitized by the parental generation EmF0 maintained on BtHC. In contrast, there was no difference in *E. mundus* parasitism between the parasitoid generations tested on BtHR. How can we explain this result? Parasitoid populations show strong adaptive potential to the defence genetic mechanisms of their hosts [51,90] and are also able to evolve virulence traits against defensive symbionts [52]. This evolutionary process can occur very quickly, within a few generations, when parasitoids are exposed to continuous selection by symbiont-infected hosts [91,92,93]. Parasitoids that evolve counter-defences against host resistance can potentially incur fitness costs [50,94]. However, there is only limited evidence [91,92] that parasitoid adaptation to symbiont-mediated defences is associated with a reduction in parasitoid fitness. In our case study, we may assume that genetic variation for virulence exists in *E. mundus* and that the parasitoid strain which was used to start our experiment was a mix of genotypes with different abilities to parasitize the two strains of *B. tabaci*, BtHC, and BtHR. Assuming a fitness cost associated with the expression of *E. mundus* counter-defences to the resistance mechanisms of the BtHC strain, rearing *E. mundus* on the BtHR strain would lead to an increase in the frequency of the parasitoid genotypes that are less virulent on BtHC and with less costs (i.e., with higher fitness) on BtHR over generations. Consequently, after *E. mundus* was reared for four and seven generations on BtHR, the lower frequency of parasitoid genotypes virulent on BtHC may have resulted in an increased susceptibility of *E. mundus* to the defensive response of BtHC nymphs. Our results provide limited evidence of fast coevolutionary responses between the host and parasitoid and of how the effect of the host strain (due to genetic background and/or defensive symbionts) can generate a selective response in the parasitoid population in favour of virulent genotypes that maximize the parasitoid fitness. Similarly, our data suggest that, in virulent populations of parasitoids, subjected to a loosening of the selective pressure that favours the host infection by defensive symbionts (akin to the reduction in the prevalence of *Cardinium* in the absence of heat stress [26,30,73]), the evolutionary response of parasitoids may be similarly rapid in the opposite direction, as less-virulent genotypes will be favoured over the virulent ones with fitness cost.

Our results lay the basis for further studies aimed at disentangling the role of genetic background and of the facultative endosymbiont *Cardinium* in the resistance of *B. tabaci* to parasitoid attack, which would be critical for better understanding the evolutionary ecology of insect hosts and parasitoids and would also inform the implementation of biological control programmes against *B. tabaci*.

## Data Availability

All relevant data are within the paper. Additional data can be supplied upon request.
